# Clinical Cases of Continued Fever

**Published:** 1869-01

**Authors:** N. S. Davis

**Affiliations:** Professor of Principles and Practice of Medicine in Chicago Medical College, and of Clinical Medicine in Mercy Hospital


### CLINICAL CASES OF CONTINUED FEVER.—IMPORTANT THERAPEUTICAL ITEMS.—THE VALUE OF STRYCHNINE IN CERTAIN PATHOLOGICAL CONDITIONS.

By N. S. DAVIS, M.D., Professor of Principles and Practice of Medicine in Chicago Medical College, and of Clinical Medicine in Mercy Hospital.

CASE I. A. B., aged about 22 years, was admitted to the medical wards of Mercy Hospital, October 17th, 1868. He was reported to have been sick in bed one week, but we could learn nothing reliable, concerning either his symptoms or treatment, during that time. At the time of admission, his expression of countenance was dull; the surface, especially of the face, hands, and neck, was suffused with a dark, dingy redness, dry, and temperature moderately increased; pulse 120 per minute, quick, and weak; lips and mouth dry; tongue covered with a thick coat, dry and brown along the middle of the dorsal surface; abdomen moderately distended, tympanitic, and presenting a few small, red papules on its surface. He was reported to have had five or six thin intestinal evacuations per day, for two or three days past. The chest was resonant and natural, except dry bronchial râles over both sides. The mental faculties dull, drowsy, and wandering, so much so as to render him incapable of giving any reliable intelligence. His muscular movements were unsteady and tremulous.

He was directed to have two or three tablespoonsful of sweet-milk and wheat-flour porridge every two hours for nourishment, with whey, bread-water, or milk and water for drink; and a teaspoonful of the following emulsion every three hours:—

℞. Ol. Terebinth., ---------- ------------ ʒiij.

Tinct. Opii, -------------------------- ʒiij.

Pulv. G. Arabic, }

White Sugar, } āā, --------------- ʒiij.

Rub together, and add Mint Water, -- ℥iij.

Mix.

He continued this treatment two days, during which time his bowels remained quiet; but his urine passed involuntarily, and he continued to exhibit all the symptoms of a strongly-marked typhus condition. The abdomen was full and tense, the pulse very soft and frequent, and the mind somnolent and muttering.

On the evening of the 19th, an enema of warm water was administered, which was followed by a moderate evacuation of fæcal matter and some flatus. During the latter part of the night he had another large evacuation in bed, which was thin and freely intermixed with blood, and the urine continued to pass without his notice. At the clinic hour on the 20th, the whole cutaneous surface was dingy, with slowness of capillary circulation; the mouth and tongue very dry, with constant tendency to gather dark sordes on the lips and teeth; mind very somnolent and muttering; respiration slow and irregular, with sharp, dry, bronchial râles; pulse 128 per minute, and weak; but less distention of the abdomen than previously. It was evident that the depression of the excito-motory nervous centres, as indicated by the slow and irregular respiration, feeble circulation, and relaxation of the sphincters, was directly threatening the life of the patient; while the copious intestinal discharge, largely intermixed with dark blood, indicated a condition of the mucous membranes scarcely less critical.

To counteract, as far as possible, the first of these pathological conditions, the patient was directed to have a teaspoonful of the following formula every four hours:—

| | | |
|---|---|---|
| ℞. | Strychnine, | 1 gr. |
| | Nitric Acid, | ʒj. |
| | Tinct. Opii, | ʒiij. |
| | Simple Syrup, } āā, | ℥iss. |
| | Water, } | |

Mix.

To aid the mineral acid and opium in restraining the further intestinal discharges, the emulsion of oil turpentine and tincture of opium was continued, in doses of a teaspoonful between each of the doses of the solution containing the strychnine. For nourishment, he was fed regularly two or three tablespoonsful of sweet-milk and wheat-flour porridge every hour, sometimes exchanging it for the same quantity of beef-tea, well salted. No further intestinal evacuations occurred, and after about 36 hours he ceased to dribble his urine in bed, and voided it regularly.

After he had followed this treatment punctually for three days, he became less somnolent, and exhibited less muttering and subsultus, but his skin remained dry; pulse soft, frequent, and weak; tongue dry, with dark sordes on the lips and teeth; bronchial rhonchi over both sides of the chest, with dulness over the lower and posterior parts; and abdomen tympanitic.

The same treatment was continued, with the addition of a warm-water enema, which procured a slight movement of the bowels, until the 27th of October. During the 25th, 26th, and 27th, the patient gradually passed from his state of somnolency and muttering to that of morbid vigilance or constant wakefulness, with less subsultus, and a slight improvement in the condition of the mouth and tongue, but the mind still wandering; pulse soft, weak, and frequent; and commencing bed-sores over the sacrum and trochanters. Thinking that the change from mental drowsiness to constant wakefulness might be the result of the continued action of the strychnine on the nervous centres, directions were given to have the interval between the doses extended to six hours, and fifteen grains of bromide of ammonium to be given at bedtime; all other directions the same as before.

On the 28th, it was found that the bromide had failed to procure sleep, although the dose was repeated a second time, and the patient appeared in all respects more exhausted and unfavorable than on the day previous. The emulsion and the strychnine solution were again given, at intervals of four hours, or two hours apart; and instead of trying further the bromide to overcome the morbid vigilance, fifteen drops of chloroform were added to each dose of the emulsion. The nourishment to be continued as before.

This treatment was continued until November 1st, with a very gradual but marked improvement in the condition of the patient.

The febrile heat had diminished; the skin was better color; sordes gathered less rapidly on his lips and teeth; the edges of the tongue were moist, though the middle was still dry and fissured; the mind less wandering, with intervals of quiet sleep. The bowels had not moved except by means of a warm-water enema, which had been given about once in three or four days. The last enema was followed by an evacuation of firmly-consistent, healthy-looking fæces. The strychnine solution was still continued every four hours, but six grains of Dover's powder and four of pulverized gum camphor were given instead of the emulsion of turpentine, etc.

Two days later, November 3d, it was found that the symptoms of convalescence had vacillated, being much more prominent every alternate day, and two grains of sulphate of quinine were given between the doses of strychnine solution, while the Dover's powder and camphor were limited to a single dose at night. This treatment was continued until May 9th, when convalescence was fully established. The bed-sores over the sacrum and trochanters had been treated by an application of the tincture of the chloride of iron, daily, and were improving; but the patient was very feeble.

The solution containing strychnine and nitric acid was continued every six hours, with the quinine between.

On the 11th, the solution was restricted to a teaspoonful three times a day, and the quinine to twice a day. From this time the patient gained rapidly, until his recovery was complete.

Case II. Mr. R., aged 25 years, was admitted into the hospital in the early part of November. About six days previously, he had been attacked with a chill, so decided as to make him think he had the commencement of an intermittent. He had felt unwell during the preceding three or four days. When the chill occurred, he called a physician, who gave some powders, and followed them by a cathartic. The latter operated freely, and during the succeeding three days he had from twelve to fifteen intestinal discharges per day.

It was at the end of these three days that he was brought to the hospital. On examination, it was found he was laboring under all the symptoms of continued fever, with great exhaustion.

He was first given the emulsion of oil of turpentine and tincture of opium, in doses of a teaspoonful every three hours, until the intestinal discharges should be stopped; with milk and flour porridge for nourishment. The next day his bowels had become quiet, but he presented every symptom of profound typhus:—countenance dull; color of skin brown or dingy; capillary circulation feeble; pulse 124 per minute, soft and weak; abdomen slightly tympanitic, and bowels quiet; some subsultus, and constant delirium; and once hemorrhage from nose. A slight papular eruption was discernible over his chest and abdomen. He continued the emulsion three times a day, and in addition took a teaspoonful of the solution of strychnine, nitric acid, and tincture of opium every four hours, same proportions as already stated in the preceding case; and the same strict attention to the giving of bland nourishment.

The further increase of prostration was arrested in twenty-four hours, but the same treatment was continued, without change, for one week; during which time, a slow but steady improvement took place.

The emulsion was then discontinued; the strychnine solution continued every six hours, with two grains of quinine alternated with it, and convalescence was fully established at the end of the second week after admission into the hospital.

CASE III. Mr. H., native of Ireland, aged 27 years; laborer; had been sick four weeks before admission into the hospital. At present, countenance dull; skin dry, congested, above the natural temperature; lips thin, retracted, and teeth covered with dark sordes; tongue covered with a dark-brown, dry coat, fissured; mouth dry; mind dull and wandering; much subsultus, the hands trembling constantly, and the tongue so tremulous that he could neither run it out nor speak plainly. His urine dribbled in bed, and a somewhat extensive superficial bed-sore existed over the sacrum. The abdomen was neither distended nor tympanitic, but he had from one to three thin fæcal evacuations daily. Pulse 130 per minute, soft and weak; respiration accelerated, but no thoracic dulness or bronchial râles.

In this case, impairment of the functions of the nervous centres and the general exhaustion were so prominent, that the patient was given at once the same strychnine and acid solution used in the other cases, in doses of a teaspoonful every four hours; five grains each of pulv. Doveri and pulv. g. camph. at night; and the prompt attention to nourishment, consisting of sweet-milk and wheat-flour porridge, and beef-tea salted with chlorate potassa, given in small but frequently repeated doses.

On the third day, he had so much improved that he could speak plain, protrude his tongue readily, and there was but little tremor of the extremities. The strychnine solution was then given only once in six hours, and a powder consisting of sulph. quinine 2 grs., pulv. Doveri 5 grs., pulv. g. camph. 3 grs., was given alternately with it; with same attention to diet as before. He continued steadily to improve, and in eight days after his admission into the hospital (five weeks from the commencement of his fever), his convalescence was fully established.

These cases strikingly illustrate a most important stage in the progress of the more severe cases of continued fever. It is a stage in which, in addition to the ordinary deterioration of the blood, and more or less local changes in the abdomen and chest, we have great depression or failure in the functions of the nervous centres; as indicated by the soft, weak pulse, the muscular tremors, the relaxation of the sphincters, etc. To counteract this condition, we have found no remedy equal in value to strychnine in solution with nitric acid, as given in the preceding cases. Tincture of opium is added, whenever the intestinal discharges are thin, or too frequent.

